# Decoding the SCFA-CpxAR-OMP axis as a dietary checkpoint against antimicrobial resistance transmission across gut-environment interfaces

**DOI:** 10.1093/ismejo/wraf156

**Published:** 2025-07-30

**Authors:** Rong Tan, Yuanyuan Song, Jing Yin, Danyang Shi, Haibei Li, Tianjiao Chen, Yating Wang, Min Jin, Junwen Li, Dong Yang

**Affiliations:** Tianjin Key Laboratory of Risk Assessment and Control for Environment and Food Safety, State Key Laboratory of Pathogen and Biosecurity, Military Medical Sciences Academy, No. 1 Dali Road, Tianjin 300050, China; Tianjin Key Laboratory of Risk Assessment and Control for Environment and Food Safety, State Key Laboratory of Pathogen and Biosecurity, Military Medical Sciences Academy, No. 1 Dali Road, Tianjin 300050, China; Tianjin Key Laboratory of Risk Assessment and Control for Environment and Food Safety, State Key Laboratory of Pathogen and Biosecurity, Military Medical Sciences Academy, No. 1 Dali Road, Tianjin 300050, China; Tianjin Key Laboratory of Risk Assessment and Control for Environment and Food Safety, State Key Laboratory of Pathogen and Biosecurity, Military Medical Sciences Academy, No. 1 Dali Road, Tianjin 300050, China; Tianjin Key Laboratory of Risk Assessment and Control for Environment and Food Safety, State Key Laboratory of Pathogen and Biosecurity, Military Medical Sciences Academy, No. 1 Dali Road, Tianjin 300050, China; Tianjin Key Laboratory of Risk Assessment and Control for Environment and Food Safety, State Key Laboratory of Pathogen and Biosecurity, Military Medical Sciences Academy, No. 1 Dali Road, Tianjin 300050, China; Tianjin Key Laboratory of Risk Assessment and Control for Environment and Food Safety, State Key Laboratory of Pathogen and Biosecurity, Military Medical Sciences Academy, No. 1 Dali Road, Tianjin 300050, China; Tianjin Key Laboratory of Risk Assessment and Control for Environment and Food Safety, State Key Laboratory of Pathogen and Biosecurity, Military Medical Sciences Academy, No. 1 Dali Road, Tianjin 300050, China; Tianjin Key Laboratory of Risk Assessment and Control for Environment and Food Safety, State Key Laboratory of Pathogen and Biosecurity, Military Medical Sciences Academy, No. 1 Dali Road, Tianjin 300050, China; Tianjin Key Laboratory of Risk Assessment and Control for Environment and Food Safety, State Key Laboratory of Pathogen and Biosecurity, Military Medical Sciences Academy, No. 1 Dali Road, Tianjin 300050, China

**Keywords:** ARGs, SCFAs, horizontal gene transfer, CpxAR-OMP pathway, gut microbiota

## Abstract

The transmission of environmental-originated antibiotic resistance genes (ARGs) into the human gut via the food chain or water has transformed the intestinal tract into a critical reservoir and dissemination hub for ARGs. Moreover, human to human oral-fecal transmission is likely to intensify this dissemination cycle. Gut microbiota harboring ARGs not only drive clinical infections but also exacerbate diverse pathologies, including inflammatory bowel disease and metabolic disorders. Furthermore, amplified ARGs can re-enter environmental compartments through fecal discharge, establishing a persistent bidirectional “gut-environment” resistance transmission cycle. In this study, we demonstrate that short-chain fatty acids (SCFAs), key metabolites derived from gut microbiota, potently suppress the horizontal transfer of ARGs. A high-fiber diet reshaped gut microbial composition, elevating SCFA production by 1.6-fold and reducing ARGs dissemination rates by up to 5.8-fold *in vivo*. The anti-conjugation activity of SCFAs was further validated through *in vitro* observations and *in vivo* models. Mechanistically, we propose the CpxAR-OMP pathway as a previously uncharacterized regulatory axis, wherein SCFAs inhibit ARGs transfer by downregulating conjugation-associated promoters (*trfAp* and *trbBp*) and disrupting membrane function via CpxAR-mediated suppression of OMPs expression. To our knowledge, this work provides comprehensive evidence of SCFAs in curbing exogenous ARGs dissemination within the gut ecosystem, deciphers the CpxAR-OMP-driven molecular mechanism, and proposes dietary fiber intervention as a feasible strategy to mitigate antimicrobial resistance across the “One-Health” continuum.

## Graphical Abstract

**Graphical Abstract:**
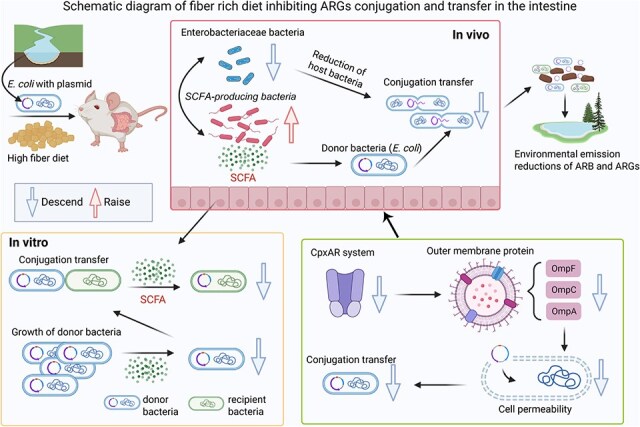

## Introduction

The global overuse of antibiotics has precipitated a critical rise in antibiotic-resistant bacteria (ARB) and ARGs across ecosystems, creating substantial risks for human and animal health [1, 2]. ARGs have now been ubiquitously detected in diverse ecosystems, with particularly high concentrations observed in aquatic environments [3, 4]. Within these microbial-rich systems [5], horizontal gene transfer (HGT) facilitates ARGs dissemination into drinking water supplies [6], tap water systems [7], and agricultural products. Compounding this issue, manure-based fertilization and wastewater irrigation practices have been shown to amplify antibiotic resistance in crop-associated microbiomes, with ARGs detected in edible plants such as lettuce and strawberries. Beyond the food chain transmission, the human-to-human oral-fecal route serves a crucial vector for the dissemination of ARB and ARGs [8–10]. These findings collectively underscore the persistent human exposure to antimicrobial resistance through multiple pathways, including contaminated food and water sources, as well as direct human-to-human transmission.

Both human-to-human and foodborne transmission sources of ARB and ARGs warrant particular concern, as they represent direct exposure pathways that can significantly impact public health. As emerging environmental contaminants, ARGs possess unique mobility through plasmids and transposons, enabling genetic exchange. Microbial-dense environments like the human gut microbiome provide ideal conditions for such transfers [11]. The constant temperature, rich nutrients, and large number of bacteria in the intestines create favorable conditions for the spread of ARGs. In vivo models have clearly demonstrated that plasmid conjugation occurs in the gut microbiome, and it can occur inter- or intraspecies [12]. Moreover, the reproduction of ARG-carrying microorganisms can facilitate vertical gene transfer, amplifying the risks of resistance transmission. Therefore, the intestines have become a “melting pot” for ARGs exchange and evolution, further highlighting the need for comprehensive research and develop effective strategies to address this growing threat to public health.

The unrestricted exchange of ARGs harbored by the gut microbiota among commensal bacteria, opportunistic pathogens, and pathogenic bacteria accelerate the rapid evolution of multidrug-resistant strains, compounding risks of opportunistic infections. Opportunistic pathogens like *Escherichia coli*, and *Klebsiella pneumoniae*, are becoming increasingly challenging to treat because of the growing antimicrobial resistance (AMR) [13]. Such genetic exchange among pathogenic bacteria poses significant threats to human health, while also establishes persistent transmission chains through the “gut-environment” cycle. Consequently, there is an urgent need to prevent the spread of ARGs among intestinal microbiota, which is essential for further enhancing human health and reducing the ARGs emission into the environment through feces. Although emerging technologies like CRISPR-based systems [14] and fecal microbiota transplantation [15] show therapeutic potential, their long-term biosafety profiles—particularly regarding human toxicity and environmental impacts—remain inadequately characterized. Consequently, this knowledge gap underscores the urgent need to develop distinct intervention strategies targeting ARG-harboring gut microbiota. Such approaches could effectively disrupt the “gut-environment” cycle, thereby offering a sustainable solution to this pressing public health challenge.

Dietary habits profoundly influence the composition and functional dynamics of gut microbial communities [16, 17]. Accumulating evidence highlights that nutritional intake can modulate the intestinal antibiotic resistome by altering microbiota profiles [18]. Comparative studies in livestock revealed diet-dependent patterns of resistance gene distribution, with concentrate-fed cattle exhibiting greater ruminal ARGs diversity and abundance than forage-fed animals [19]. This observation is consistent with our previous research, which showed that high-sugar, high-fat, and high-protein diets can considerably promote the spread ARGs among the intestinal microbiota of mice [20]. Human observational data further support this link, as U.S. population analyses associate fiber-rich diets with reduced intestinal ARGs loads [21]. Mechanistically, dietary fibers drive microbial restructuring, metabolic reprogramming, and microenvironmental shifts [22], collectively creating conditions unfavorable for ARGs transfer. Unlike pharmacological interventions, dietary modulation represents a natural, biocompatible strategy to suppress resistance transmission while promoting ecosystem-wide microbial balance.

SCFAs, including acetate, propionate, and butyrate, are microbial metabolites produced through colonic fermentation of dietary fiber by anaerobic symbionts. These molecules serve dual roles as energy substrates for intestinal epithelial cells and modulators of gut defense mechanisms. By acidifying the intestinal lumen, SCFAs suppress pathogen colonization while selectively promoting commensal microbial adhesion. Moreover, butyrate regulates intestinal homeostasis through PPAR-γ-mediated suppression of inducible nitric oxide synthase, reducing nitrate availability and curbing *Enterobacteriaceae* proliferation [23]. High-fiber diets amplify SCFA production, fostering microbial ecosystem stability, and reinforcing the intestinal mucosal barrier [24]. Therefore, a favorable intestinal microenvironment modulated by a fiber-rich diet may limit the spread of ARGs, providing a new avenue in the search for effective dietary interventions against the spread of ARGs. However, the molecular interplay among SCFAs, bacterial conjugation machinery, and host–microbe interactions remain unclear. The study presented comprehensive overview of the effects of SCFAs on the dissemination of ARGs in the gut which not only contributes significantly to human health but also effectively curtails the release of ARGs into the environment. This holistic approach, in line with the “One Health” concept (Interconnectedness of human, animal, and environmental health), is essential for addressing the complex challenges that affect the well-being of all components within our shared ecosystem.

## Materials and methods

### Animal experiments

Experiment 1 (Dietary Intervention and ARB Exposure): 6-week-old male SPF BALB/c mice (Beijing Weitonglihua) were acclimated to experimental diets for 1 week prior to baseline fecal collection. Antibiotic susceptibility was initially assessed by measuring inhibition zone diameters using commercial antibiotic discs (Beijing San Yao), which provided preliminary characterization of gut microbiota resistance profiles. Subsequently, conjugative plasmids harbored by the gut microbiota were identified through agarose gel electrophoresis analysis. Following randomization, forty animals were equally divided into two dietary cohorts (n = 20 per group): a high-fiber diet group (15% fiber content) [25] and a standard chow control group, maintained for 8 weeks under controlled housing conditions (single cage per mouse with ad libitum access to food and water). Post-intervention analysis confirmed the successful induction of distinct gut microbiota profiles between dietary groups. Subsequently, mice from each dietary regimen were further stratified into experimental (ARB-gavaged, n = 10) and control (saline-gavaged, n = 10) subgroups for downstream investigations. Daily oral administration of 100 μl ARB suspension (10^9^ CFU) or saline continued for 7 days. The ARB construct comprised an mCherry-expressing *E. coli* isolate from murine fecal microbiota, engineered to carry an environmental RP4 plasmid encoding GFP, tetracycline resistance, and kanamycin resistance [20]. Ad libitum feeding was maintained throughout the study with weekly weight monitoring. Serial fecal samples were collected at predetermined intervals and cryopreserved for downstream analyses.

Experiment 2 (SCFAs metabolite validation): After one-week acclimatization, six-week-old male SPF BALB/c mice were randomly allocated into five groups: four experimental groups and one control group (n = 10 per group). Four experimental cohorts received daily oral administration of 100 μl solvent oil containing distinct butyrate derivatives: (4-methoxyphenyl) butyric acid, 4-hydroxybutyric acid, 3-hydroxy-3-methylbutyric acid, or 4-(4-chlorophenyl) butyric acid. Control animals received solvent-only vehicle under identical dosing conditions (100 μl/day, 4 weeks). Post-treatment, all subjects underwent 7-day ARB challenge (100 μl/day) matching Experiment 1 protocols.

All the experimental procedures were approved by the Ethics Committee for the Welfare of Experimental Animals of Military Medical Sciences Academy. The experiments were conducted in strict accordance with the guiding principles of animal research set by the Chinese Physiological Society.

### Molecular profiling of conjugation regulators

DNA was extracted from fecal samples using a fecal genome extraction kit (QIAGEN, USA). Primers were designed according to the specific gene tag fragment *traG* on the RP4 plasmid. For the absolute quantification of gene copy numbers, the cycle threshold values obtained from fluorescence-based quantitative PCR were input into the formula derived from the standard curve of plasmid DNA. For the assessment of gene regulation, total RNA was extracted from fecal samples using a total RNA extraction kit (QIAGEN, USA). Subsequently, the extracted RNA was reverse transcribed into complementary DNA using a reverse transcription kit (TaKaRa, Japan). To detect and statistically analyze the regulatory genes *trfAp* and *trbBp* related to conjugation and DNA transfer, specific primers were designed, and fluorescence-based quantitative PCR was performed as described in the literature [12]. Gene expression levels were normalized to the bacterial 16S rRNA housekeeping gene to control for variations in bacterial density and gene copy number.

### Metagenomics analysis of intestinal ARGs

Following fecal DNA extraction, sample purity and concentration were verified using a NanoDrop 2000 spectrophotometer (Thermo Fisher Scientific, USA). Subsequently, short-insert libraries (350–500 bp) were prepared using the NovaSeq X Plus platform (Illumina, USA). Only libraries passing stringent quality control were pooled according to their effective concentrations and target sequencing depth, followed by paired-end sequencing (PE150) with an average output of 10–20 Gb per sample. Raw sequencing data underwent quality control through readfq (v8) preprocessing to obtain high-fidelity sequences [26]. SOAPdenovo-assembled scaftigs from individual and pooled samples were filtered to retain fragments ≥500 bp [27]. MetaGeneMark (v2.10) predicted open reading frames (ORFs) in qualifying scaftigs, with subsequent exclusion of ORFs <100 nt [28]. Processed sequences were aligned against the initial gene catalog using Bowtie2, eliminating genes with ≤2 mapped reads per sample [29]. Gene abundance quantification incorporated read counts normalized by sequence length. Final annotation against the Comprehensive Antibiotic Resistance Database employed Resistance Gene Identifier (RGI) software [30], enabling relative ARGs abundance calculation and resistance profile mapping through integrated RGI outputs and gene abundance data.

### Determination of intestinal metabolites

Fecal samples were cryogenically homogenized in liquid nitrogen and extracted with 500 μl ice-cold 80% methanol/0.1% formic acid solution. After vortex-mixing and 5-min ice incubation, debris was pelleted by centrifugation (15 000 g, 4°C, 10 min). The supernatant was diluted with MS-grade water to 53% methanol concentration and re-centrifuged under identical parameters. Processed supernatants were subjected to LC–MS analysis [30], with blank controls using 53% methanol/0.1% formic acid undergoing parallel processing. Raw LC–MS data were aligned using retention time (±0.2 min) and mass accuracy (±5 ppm) thresholds. Peak integration and molecular formula prediction utilized mzCloud database matching, followed by blank-subtracted background correction and area normalization. This workflow enabled precise metabolite identification and quantification, elucidating intestinal metabolic profiles.

### *In vitro* conjugation assay and bacterial growth kinetics analysis

Conjugation assays employed two donor strains (*E. coli*-RP4-Tet^R^Km^R^ and *E. coli*-lacIq-RP4-Tet^R^Km^R^) paired with receptor strains *E. coli*-Cl^R^ and *Kp*-NDM-1^R^. All four bacterial strains were cultured overnight in LB medium at 37°C with shaking. Bacterial growth was monitored until reaching mid-exponential phase (OD600 = 1.5 for *E. coli* at and 0.8 for *Kp*). cells were then harvested by centrifugation (6000 rpm, 25°C, 5 min), washed three times with PBS to remove residual antibiotics, and finally resuspended to a standardized concentration of 10^9^ CFU/ml in fresh PBS. Four donor-receptor combinations (1:1 ratio) were treated with four differentially abundant butyrate, cellulose, or DMSO (concentrations detailed in Supplementary Table 1). Mixtures were incubated at 37°C for 6–8 hr, serially diluted (10^−2^), and plated via pour-plate method using 1 ml inoculum per 15 ml agar. Solidified plates were inverted for 24 hr incubation at 37°C. For growth kinetics analysis, *E. coli* and *K. pneumoniae* inoculated in LB broth were supplemented with metabolites (200 μl/well) and monitored in a Bioscreen C system (Finland). Optical density (600 nm) was recorded every 30 min over 24 hr to generate time-resolved growth curves under test conditions.

### Macro transcriptome gene determination

RNA sequencing analysis was performed on bacterial samples from butyrate-treated conjugation assays (Supplementary M1). Total RNA was extracted and assessed for purity via agarose gel electrophoresis, followed by quantification of integrity and yield using an Agilent 2100 bioanalyzer (Agilent Technologies, USA). Libraries were constructed from qualified RNA (≥1.5 ng/μl), with insert size validation and qPCR-based concentration determination (≥2 nM effective concentration). Ten normalized libraries underwent paired-end sequencing (150 bp) on the NovaSeq X Plus platform (Illumina, USA). Raw reads were processed through a comprehensive bioinformatics pipeline: Quality-filtered data were aligned to reference genomes for transcript reconstruction and UTR/sRNA characterization. Differential gene expression analysis incorporated GO and KEGG functional annotation, complemented by SNP/Indel detection and WGCNA-based co-expression network modeling [31]. This integrated approach elucidated condition-specific transcriptional responses and regulatory networks.

### Gene knockout and replenishment

The *cpxA* and *cpxR* genes in donor (*E. coli*) were disrupted via λ-Red recombination. Homologous arms (50 bp flanking each target gene) and primers spanning the chloramphenicol resistance (Cm^R^) cassette were designed. Using plasmid pACYC184 as template, high-fidelity PCR amplified Cm^R^-flanked *cpxA*/*cpxR* fragments. Electrocompetent EcoNS cells harboring the temperature-sensitive pKD46 helper plasmid were transformed with these fragments [32]. Positive clones (EcoNS/Δ*cpxA*::Cm and EcoNS/Δ*cpxR*::Cm) were selected on Cm plates at 37°C, enabling simultaneous plasmid loss and knockout verification via PCR.

For functional restoration, *cpxA* and *cpxR* ORFs amplified from wild-type *E. coli* were cloned into XbaI/EcoRI-digested pRK415 (Tet^R^), generating complementation plasmids pRK415-*cpxA* and pRK415-*cpxR*. These constructs were electroporated into *E. coli* β2155, with transformants selected on tetracycline plates. Conjugative transfer experiments paired β2155/pRK415-*cpxA* with EcoNS/Δ*cpxA*::Cm, and β2155/pRK415-*cpxR* with EcoNS/ΔcpxR::Cm. Complemented strains were validated by PCR and sequencing (Supplementary Fig. 1).

### Statistical analysis

Statistical analyses were performed using GraphPad Prism 7.0 and RStudio. Beta diversity patterns were visualized through principal component analysis (PCA), heatmaps, and circos plots generated using R’s ggplot2 package. Group dissimilarities were statistically validated using ANOSIM, MRPP, and PERMANOVA (vegan package). Pearson correlation analysis examined metabolite-microbiota associations, while a random forest model with 5-fold cross-validation (rfcv function) identified optimal diagnostic features. Multiple testing correction employed Benjamini-Hochberg FDR (significance: FDR < 0.05). Community assembly processes were evaluated using a neutral community model, where OTU frequency-abundance relationships were fitted via R’s Hmisc and minpack.lm packages to quantify stochasticity in microbial composition.

## Results

### Dietary fiber attenuates intestinal ARGs dissemination

**Figure 1:**
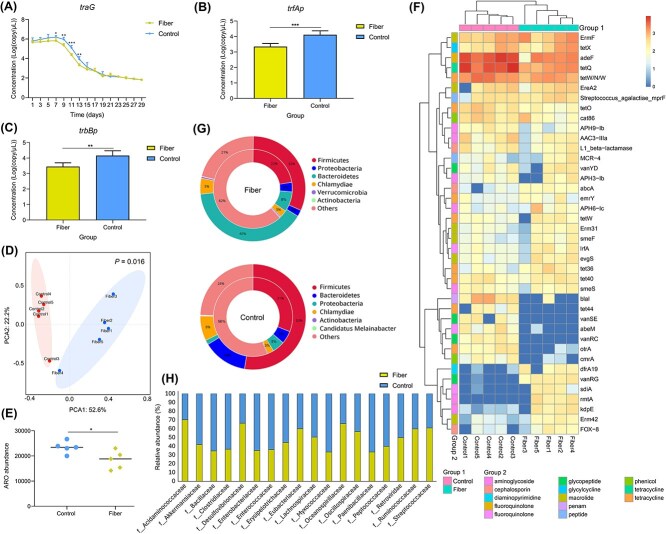
Effects of the high fiber diet on ARGs in the mice intestine. The expression levels of the target gene *traG* over time under different diets (A). The regulatory gene *trfAp* for plasmid replication (B). The regulatory gene *trbBp* formed by mating pairs (C). Based on the PCA analysis of the intestinal resistance genome at the genus level (D). Scatterplot showing two groups of known ARO number (E). Heatmap of the top 40 ARGs in each resistance group. Note on the right: group 1 represents the experimental grouping, and group 2 represents the resistance type of the resistance gene (F). Double circle diagram of distribution of total intestinal bacteria and ARGs host bacteria at the phylum level, with the outer circle representing the total intestinal microbiota and the inner circle representing the host bacteria with ARGs (G). Histogram of the host with ARGs at the family level (H).

To assess high-fiber diet impacts on intestinal ARGs dissemination, mice were fed either a high-fiber or normal diet and orally inoculated with RP4 plasmid-carrying *E. coli* to track resistance dynamics. Longitudinal fecal genomic analysis via *traG* qPCR (a conjugation regulator gene on RP4) revealed time-dependent trajectories: both groups showed initial *traG* accumulation during inoculation, followed by post-intervention decline to ~10^2^ copies. Critically, the high-fiber group exhibited significantly lower *traG* loads at Days 7, 9, 11, and 13 (*P* < .05; Fig. 1A), with three distinct phases-accumulation, decline, and stabilization. At peak accumulation, conjugation-related promoters (*trfAp*, *trbBp*) were suppressed in the high-fiber group versus controls (*P* < .05; Fig. 1B, C). These findings demonstrate that high-fiber intake curbs exogenous ARGs amplification, conjugation, and environmental release compared to standard diets.

Resistome profiling demonstrated that dietary patterns profoundly reconfigure antibiotic resistance dynamics. Multivariate analysis revealed significant β-diversity segregation between dietary cohorts (*R*^2^ = 0.32, *P* = .016; Fig. 1D), where the high-fiber group exhibited a 21.4% reduction in total ARGs abundance compared to controls (Fig. 1E). Of particular clinical relevance, genes *adeF*, *tetQ*, *blaI*, and *vanSE* showed selective attenuation (*P* < .05; Fig. 1F). This ARGs suppression correlated with phylum-level microbial restructuring: the proportion of ARG-hosting *Firmicutes* decreased significantly from 31% to 22% (*P* < .01), while *Bacteroidetes*-associated ARGs increased from 5% to 8% (*P* = .003; Fig. 1G). Mechanistically, high-fiber intervention depleted ARG-enriched taxa including *Akkermansiaceae*, *Enterobacteriaceae*, and *Lachnospiraceae* (*P* < .01; Fig. 1H). Concurrently, it suppressed *Proteobacteria* abundance (4% vs. 5% in controls) and *Enterobacteriaceae* (*P* = .007), a clinically significant finding given their established role as reservoirs for plasmids [33, 34]. Collectively, these findings establish a causal chain linking dietary modulation of gut microbiota architecture to containment of antimicrobial resistance determinants.

### Gut microbiota reshaping via fiber intervention

**Figure 2:**
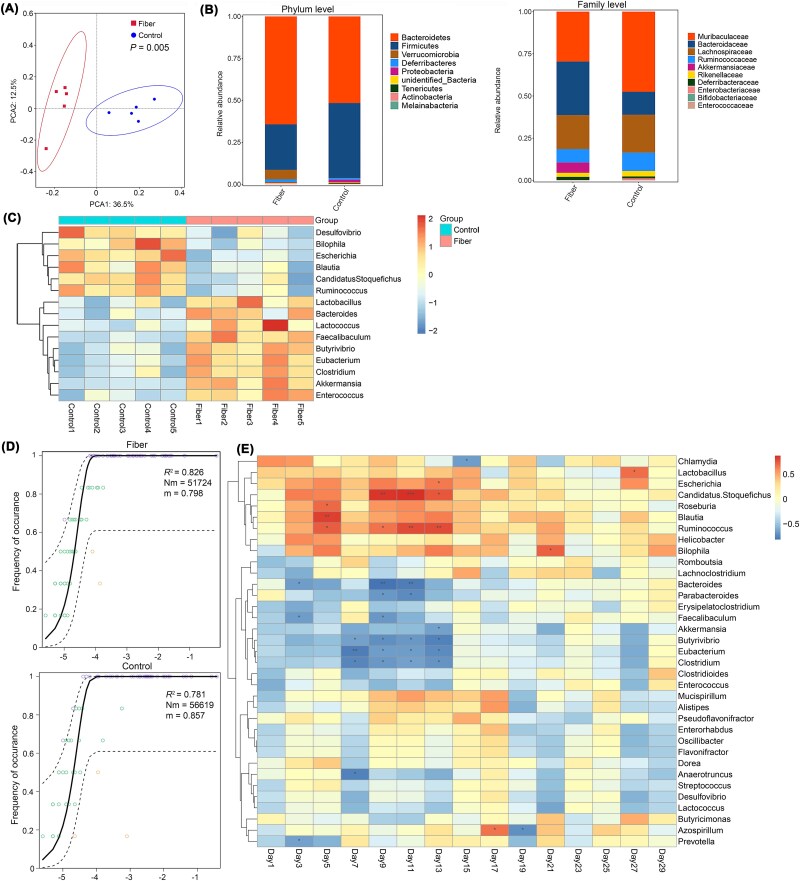
Effects of the high fiber diet on intestinal microbiota. Based on the PCA analysis of intestinal microbiota at the genus level (A). Histogram of relative abundance of top 10 taxa at the phylum and family levels (B). Heatmap of species distribution at the genus level of two groups of different bacteria (C). In the neutral model of intestinal community assembly, *R*^2^ is the influence rate of environmental factors on the community, representing the randomness of the community, N_m_ represents the diffusion degree of the community, and m represents the diffusion rate of the community (D). Correlation analysis of ARGs expression levels and significant differences in bacteria at different times (E).

To investigate how high-fiber diets suppress ARGs dissemination, we performed multi-level phylogenetic analysis of gut microbiota. Genus-level principal component analysis showed significant microbial community segregation between high-fiber and control groups (*P* = .005, Fig. 2A). The high-fiber diet elevated the *Bacteroidetes*/*Firmicutes* ratio (2.37 vs. 1.16 in controls), with increased *Bacteroidetes*, *Verrucomicrobia*, and *Deferribacteres* but reduced *Firmicutes* and *Proteobacteria*. Taxonomic profiling revealed enrichment of *Bacteroidaceae*, *Akkermansiaceae*, and *Deferribacteraceae* families, contrasting with suppressed *Muribaculaceae* and *Enterobacteriaceae* (Fig. 2B). Beneficial genera (*Lactobacillus* spp., *Akkermansia* spp.) were enriched while ARG-associated *Escherichia* spp. and *Bilophila* spp. declined (Fig. 2C). This restructuring suggests dual suppression: fibrolytic bacteria competitively exclude ARGs carriers through niche occupation, while SCFA metabolites from *Bacteroides* spp. and *Akkermansia* spp. inhibit opportunistic pathogens.

Neutral community modeling revealed that stochastic processes dominated gut microbiota assembly in both dietary groups (*R*^2^ > 0.7). High-fiber communities exhibited enhanced stability through higher stochasticity and lower diffusion rates. Although ARB reduced stochasticity in both groups, high-fiber microbiota showed stronger resilience (Fig. 2D). Time-series analysis identified persistent negative correlations between ARGs suppression and SCFA producers (*Butyrivibrio* spp., *Eubacterium* spp.) or mucin specialists (*Akkermansia* spp.) (Spearman’s ρ < −0.65, FDR < 0.05; Days 3–13). Conversely, ARGs accumulation correlated positively with *Escherichia* spp. and inflammatory taxa (ρ > 0.60, q < 0.05; Fig. 2E). These dynamics confirm that fiber-driven microbial remodeling establishes ecological barriers against ARGs spread via niche competition and metabolite inhibition.

### Fiber modulates SCFAs biosynthesis

**Figure 3:**
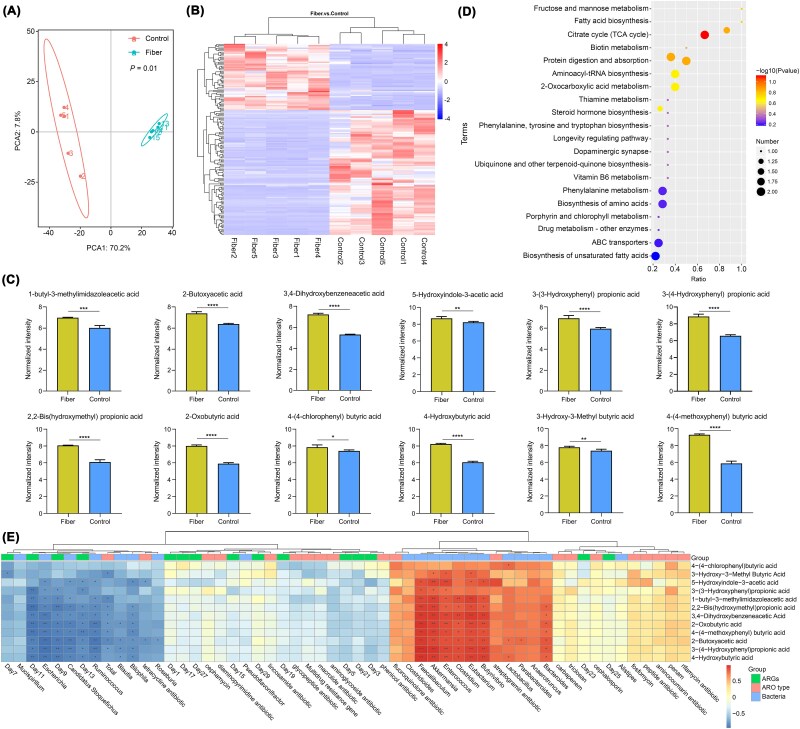
High fiber diet affected intestinal metabolites. PCA analysis of metabolites in each group (A). Heatmap of differential metabolite clustering (B). Comparison histogram of SCFAs between the two groups (C). The enrichment bubble diagram of KEGG metabolic pathway (D). Correlation analysis between the number of ARGs, the number of different bacteria, the number of intestinal resistance types and SCFAs (E).

Gut microbiota structural remodeling induced by dietary intervention was accompanied by profound metabolic reprogramming. Metabolomic profiling revealed distinct fecal metabolite signatures between high-fiber and control groups, with hierarchical clustering and principal component analysis confirming diet-driven separation (Fig. 3A, B). The high-fiber group exhibited elevated concentrations of SCFAs, including four acetate, three propionate, and five butyrate derivatives (*P* < .05; Fig. 3C). Pathway enrichment analysis identified 20 significantly altered metabolic routes, seven of which directly regulated SCFA biosynthesis and metabolism: TCA cycle, thiamine-dependent carboxylation, biotin metabolism, and fatty acid biosynthesis pathways (Fig. 3D). These findings demonstrate that high-fiber diets enhance microbial SCFA production through coordinated activation of cross-feeding networks and metabolic cascades.

Temporal correlation analysis uncovered dynamic relationships between SCFA levels and antimicrobial resistance dynamics. SCFA concentrations showed persistent negative correlations with exogenous ARGs transfer frequencies across multiple time points (Days 3–27), reaching statistical significance on Days 7–13 (*P* < .05). Metabolite-microbiota interactions revealed dual modulation: SCFAs positively correlated with beneficial taxa (*Akkermansia* spp., *Lactobacillus* spp., *Butyrivibrio* spp.) while negatively associating with ARG-harboring opportunists (*Escherichia* spp., *Bilophila* spp.) and multidrug resistance determinants (*P* < .05; Fig. 3E). The inverse correlation between SCFA abundance and ARGs dissemination suggests a metabolite-mediated containment mechanism. Butyrate and propionate may suppress HGT by modulating bacterial conjugation efficiency. Furthermore, SCFA-driven proliferation of commensal taxa likely establishes colonization resistance against ARB through niche exclusion. These findings position dietary fiber as a dual modulator of gut metabolomics and resistome dynamics, offering a nutritional strategy to counteract antimicrobial resistance.

### SCFAs mediated ARGs suppression *in vivo*

**Figure 4:**
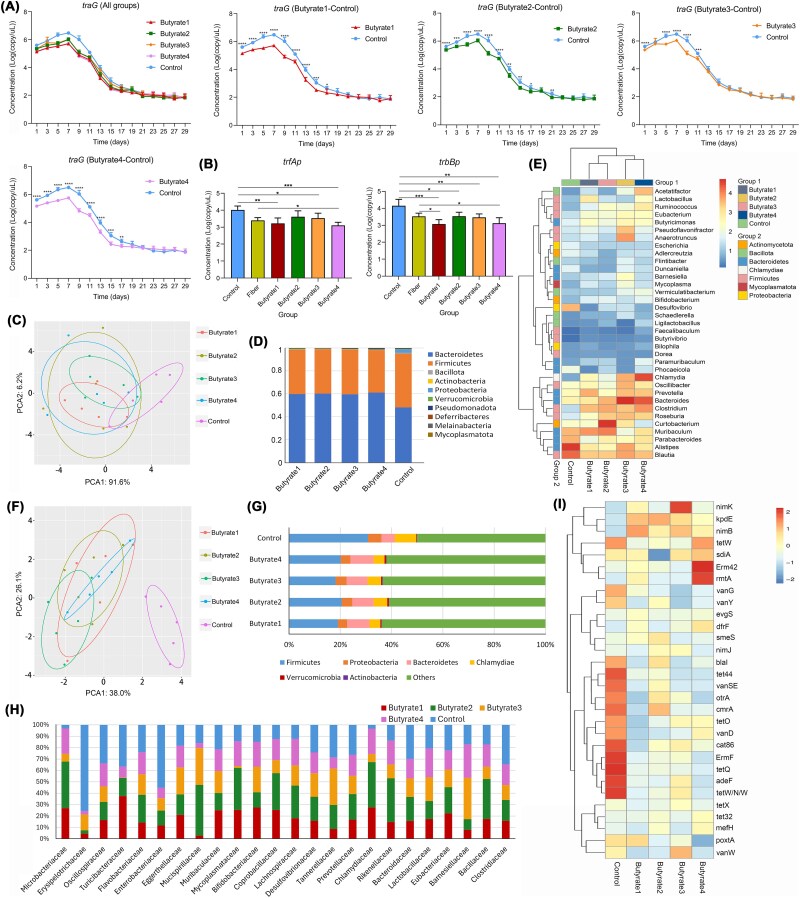
Effects of SCFAs on intestinal microbiota and host bacteria of ARGs in mice (n = 5). Changes in target gene expression over time under different SCFAs (A). Expression of regulatory genes for *trfAp* and *trbBp* under SCFAs (B). Based on the PCA analysis of intestinal microbiota at the genus level (C). Histogram of relative abundance of top 10 taxa at the phylum and family levels (D). Heatmap of the top 35 taxa of each group at the genus level (E). Based on the PCA analysis of the intestinal resistance genome at the genus level (F). Histogram of the host with ARGs at the phylum level (G). Histogram of the host with ARGs at the family level (H). Heatmap of the top 30 ARGs in each resistance group (I). Four butyrate derivatives: Butyrate1, 4-(4-methoxyphenyl) butyric acid; Butyrate2, 3-hydroxy-3-methylbutyric acid; Butyrate3, 4-hydroxybutyric acid; Butyrate4, 4-(4-chlorophenyl) butyric acid.

Building on our previous discovery of SCFAs suppressing ARGs dissemination, we tested four structurally distinct butyrate derivatives (butyrate 1–4) as potential mediators of high-fiber diet effects. Dynamic monitoring of *traG* gene expression revealed a transient increase during gavage, followed by a gradual decline to ~10^2^ post-intervention in both butyrate and control (DMSO) groups, mirroring trends observed with high-fiber diets. All butyrate groups exhibited significantly lower *traG* levels than controls at multiple time points. For instance, butyrate 1 and 4 showed sustained suppression from Days 1–17, while butyrate 2 demonstrated inhibition extending to Day 21 (Fig. 4A). Parallel analysis of conjugation-related regulatory genes revealed marked reduced *trfAp* expression in butyrate 1, 3, and 4 groups, and significantly downregulated *trbBp* levels across all butyrate derivatives compared to controls (Fig. 4B). These results demonstrated that specific butyrate derivatives exert stronger ARGs transfer inhibition compared to whole high-fiber diets, positioning them as pivotal mediators of dietary modulation in HGT.

PCA of gut microbiota at the genus level demonstrated distinct clustering between butyrate-treated and controls (Fig. 4C). Butyrate administration elevated the *Bacteroidetes*/*Firmicutes* ratio (1.53–1.65 vs. 1.03 in controls) and increased abundances of *Bacteroidetes*, *Actinobacteria*, and *Melainabacteria*, while reducing *Firmicutes* and *Proteobacteria* (Fig. 4D). Genus-level shifts included depletion of *Alistipes* spp. and *Desulfovibrio* spp., alongside enrichment of *Lactobacillus* spp. and *Butyricimonas* spp. in butyrate groups (Fig. 4E). Corresponding resistome analysis revealed altered ARGs distribution patterns: PCA of ARGs revealed significant clustering between butyrate-treated and controls (Fig. 4F). Moreover, butyrate groups showed reduced ARGs carriage in *Firmicutes* (18%–21% vs. 31% in controls) and *Proteobacteria* (3%–4% vs. 5%), with parallel decreases in ARG-hosting families like *Enterococcaceae* (Fig. 4G, H). Control mice exhibited higher abundances of clinically relevant ARGs (*tetW*, *vanG*, *ermF*), whereas butyrate groups predominantly harbored less prevalent variants (*nimK*, *rmtA*) (Fig. 4I). Consistently, the butyrate-induced enrichment of *Lactobacillus* spp. and reduction of *Desulfovibrio* spp. align with prior studies demonstrating that *Lactobacillus* spp. competitively inhibit pathogenic bacteria and suppress conjugation through niche competition [35], while *Desulfovibrio* spp. is associated with pro-inflammatory environments favoring ARGs exchange. This restructuring of microbial communities and resistomes implies that butyrate limits ARGs dissemination by reducing host bacterial availability and altering conjugation-favorable ecological niches.

The combined evidence establishes butyrate derivatives as critical mediators of high-fiber diet effects on ARGs dynamics. Their dual action—direct suppression of conjugation machinery (*trfAp*/*trbBp*) and indirect modulation of ARGs host populations-suggests a multi-layered inhibition mechanism. The preferential reduction of *Firmicutes*, major ARGs reservoirs, coupled with diminished *Proteobacteria* abundance, likely creates an ecological barrier against resistance gene persistence.

### SCFAs directly inhibits ARGs transfer *in vitro*

**Figure 5:**
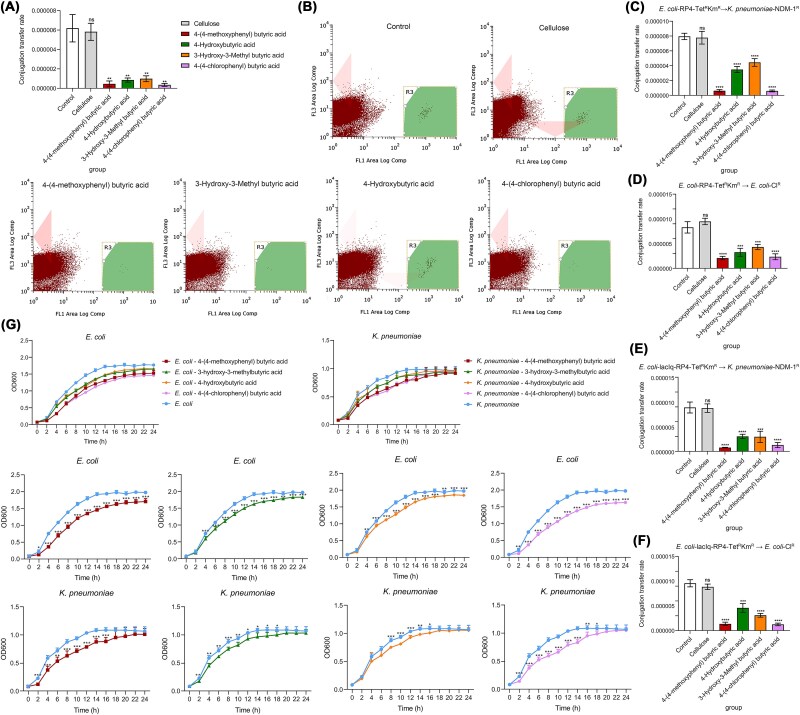
SCFAs affected the conjugative transfer of ARGs and the growth of donor and recipient bacteria. Histogram of the conjugative transfer rate of single bacterial strains to intestinal microbiota *in vitro* under the influence of different metabolites (A). Flow cytometry scatter diagram of conjugation and transfer of single bacterial strains to intestinal microbiota *in vitro* under the influence of different metabolites (B). Histogram of the *in vitro* conjugative transfer rate of different conjugation pairs of bacterial strains under the influence of different metabolites (C, D, E, F). Growth curves of donor and recipient bacteria under four SCFAs (G).

Building on the demonstrated efficacy of butyric acids in suppressing ARGs dissemination, we investigated their direct inhibitory effects on bacterial conjugation *in vitro*. Flow cytometry analysis revealed that while cellulose (a high-fiber analog) showed negligible impact on ARGs transfer (6.2 × 10^−6^ vs. control: 5.8 × 10^−6^), all tested butyric acids significantly reduced conjugation rates. 4-(4-chlorophenyl) butyric acid and 4-(4-methoxyphenyl) butyric acid exhibited strongest suppression, achieving 8.6- to 36-fold transfer reduction versus controls (Fig. 5A, B). Consistent inhibition patterns emerged across four bacterial conjugation pairs, with butyrate derivatives showing 3–8-fold suppression (*P* < .05; Fig. 5C–F). Growth curve assays further demonstrated these derivatives significantly reduced donor/recipient bacterial proliferation, correlating with their conjugation suppression. Comparative analysis revealed that among all tested compounds, the 4-(4-chlorophenyl) butyric acid and 4-(4-methoxyphenyl) butyric acid demonstrated the most potent inhibitory effects (Fig. 5G). Mechanistically, the observed reduction in bacterial cell density may directly contribute to the decreased conjugation frequency, as bacterial conjugation efficiency is known to be cell density-dependent.

### CpxAR-dependent conjugation inhibition via membrane modulation

**Figure 6:**
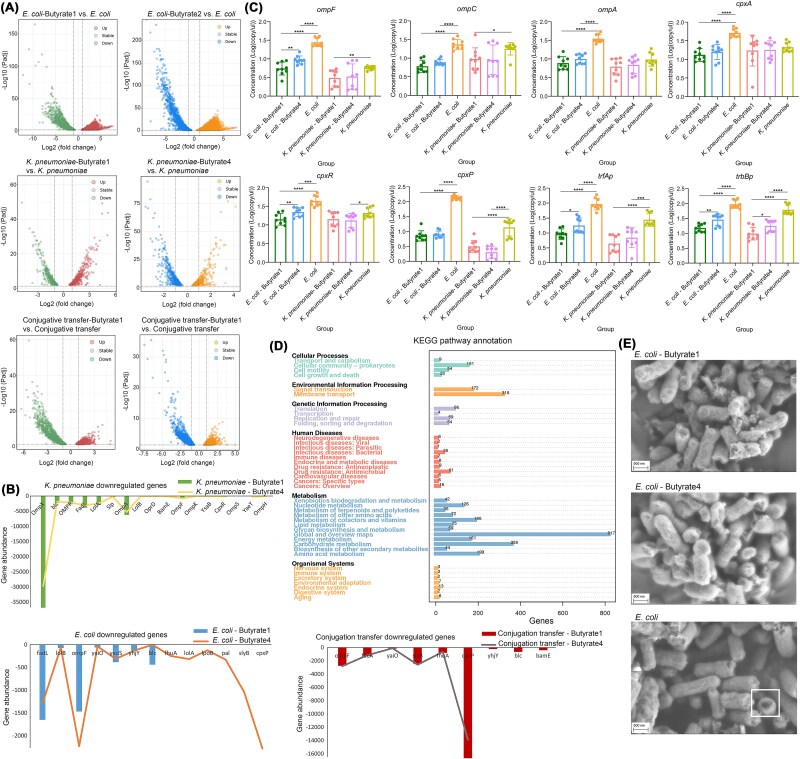
Effects of SCFAs on the expression of conjugation-related genes. Volcano plots of gene expression in donor and recipient bacteria, as well as conjugated pairs under the action of two types of butyrate (A). Genes with significantly reduced expression in donor and recipient bacteria, as well as conjugated pairs (B). Expression levels of genes related to conjugation transfer in donor and recipient bacteria (C). KEGG pathway annotation under the action of butyrate (D). SEM images of *E. coli* in two types of butyric acid, the positions circled in the white-bordered box are the pores on the bacterial cell membrane (E).

To investigate the mechanism underlying butyric acids’ suppression of bacterial conjugation, we conducted transcriptomic analyses of *E. coli*, *K. pneumoniae*, and their conjugation systems treated with 4-(4-methoxyphenyl) butyric acid (butyrate 1) and 4-(4-chlorophenyl) butyric acid (butyrate 4). Transcriptome analysis revealed marked downregulation of membrane-associated genes (*ompF*, *ompA*, *fadL*) and *cpxAR* two-component system components (*cpxR*, *cpxP*), with minimal compensatory upregulation (Fig. 6A, B). These suppressed genes predominantly were clustered in pathways critical for membrane integrity and environmental sensing. Quantitative PCR analysis revealed significant downregulation of membrane protein genes (*ompF/C/A*) and *cpxAR* signaling elements in *E. coli* following treatment with both butyric acid derivatives, with weaker effects in *K. pneumoniae* (Fig. 6C). Correspondingly, *trfAp* and *trbBp* were significantly downregulated in the donor strain (*E. coli*), aligning with reduced conjugation efficiency. This donor-specific inhibition implies that butyric acids primarily disrupt ARGs dissemination by targeting plasmid-export machinery in the originating bacterium rather than recipient cells. Functional enrichment analysis identified membrane transport as the most impacted pathway, alongside two other common modulated bacterial functional pathways: global regulation systems and carbohydrate metabolism (Fig. 6D), consistent with SEM observations of altered membrane architecture. Butyrate-treated bacteria exhibited enhanced membrane integrity compared to controls, with reduced surface irregularities that likely hinder pilus-mediated plasmid transfer (Fig. 6E). These structural changes correlate with transcriptional downregulation of pore-forming proteins (e.g. OmpF), which are critical for intercellular contact during conjugation. The coordinated suppression of OMPs and CpxAR signaling by 4-(4-methoxyphenyl) butyric acid and 4-(4-chlorophenyl) butyric acid demonstrates a dual mechanism for ARGs inhibition. Firstly, these compounds physically obstruct conjugation channels by downregulating porin expression, limiting intercellular contact essential for plasmid transfer. Secondly, they disrupt stress-responsive CpxAR signaling, impairing bacterial capacity to mobilize plasmids under environmental pressures.

### CRISPR validation of CpxAR in ARGs containment

**Figure 7:**
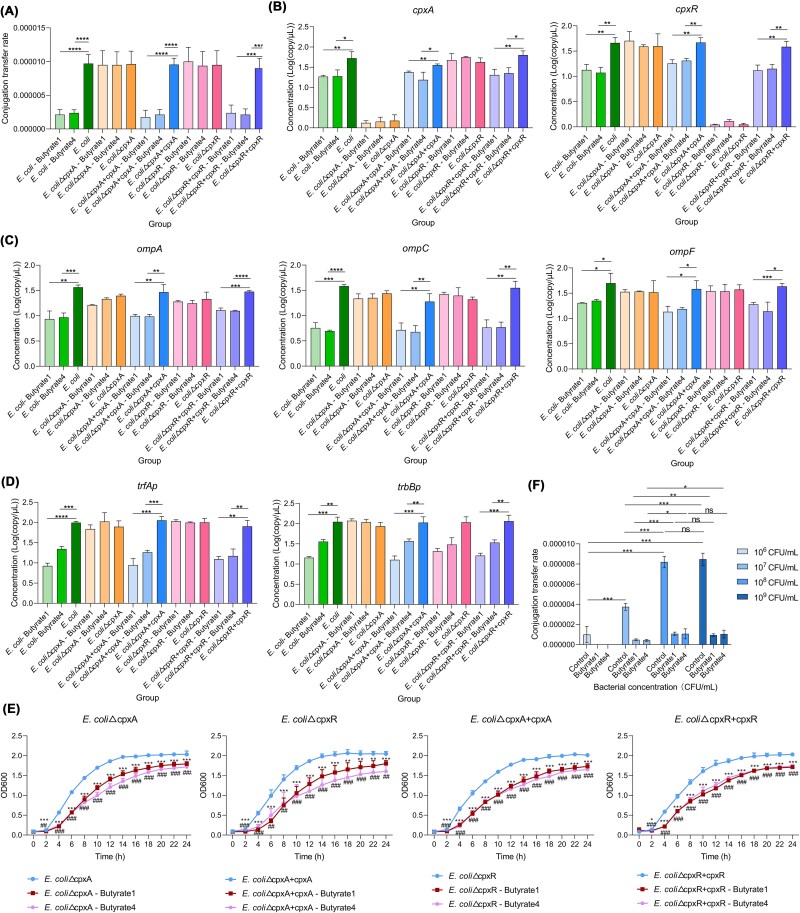
The effect of SCFAs on the expression of conjugation related genes in knockout and complement strains. The conjugation transfer rates of knockout and complement strains in two types of butyric acid (A). The *cpxA* and *cpxR* gene expression levels of knockout and complement strains in two types of butyric acid (B). The outer membrane protein gene expression levels of knockout and complement strains in two types of butyric acid (C). The expression levels of conjugation transfer regulatory genes in knockout and complement strains in two types of butyric acid (D). Growth curves of knockout and complementary strains in two distinct of butyric acid (E). Dose-dependent effect of bacterial cell density on conjugation efficiency (F).

To dissect the role of the *cpxAR* in butyrate-mediated ARGs suppression, we generated *cpxA* and *cpxR* knockout mutants in donor *E. coli*. Conjugation assays revealed that butyrate 1 and 4 failed to inhibit plasmid transfer in knockout strains, with transfer rates matching controls (Fig. 7A). Genetic complementation restored butyrate sensitivity, reducing conjugation frequencies to wild-type levels. This functional rescue confirmed *cpxAR* as the primary signaling node mediating butyrate effects on conjugation efficiency. Transcriptional analysis demonstrated near-undetectable *cpxAR* expression in knockout strains (Fig. 7B), accompanied by restored expression of outer membrane proteins (*ompF*, *ompC*, *ompA*) and conjugation regulators (*trfAp*, *trbBp*) under butyrate treatment (Fig. 7C, D). Complementation re-established butyrate-induced downregulation of these targets, mirroring wild-type responses. These findings indicate that CpxAR signaling is essential for butyrate to suppress porin expression and plasmid transfer machinery.

Beyond the observed changes in membrane protein-related genes, both knockout and complementation strains exhibited moderate growth inhibition under the two butyrate conditions, displaying a comparable decline pattern to the wild-type strain (Fig. 7E). These findings indicated that the targeted gene deletions exerted negligible effects on bacterial growth kinetics. Moreover, bacterial density significantly correlated with conjugation rates in subsequent experiments. The results showed expected density-dependent conjugation rates (10^−6^ to 10^−5^), with significant reductions at lower densities (50% decrease at 10^7^ CFU/ml and 70% decrease at 10^6^ CFU/ml compared to 10^9^) (Fig. 7F, Supplementary Fig. 2). Conclusively, butyrate treatment groups consistently exhibited >50% reduction in conjugation rates versus controls at equivalent densities, demonstrating that population density affects conjugation efficiency, whereas butyrate derivatives exert additional independent inhibitory effects.

**Figure 8:**
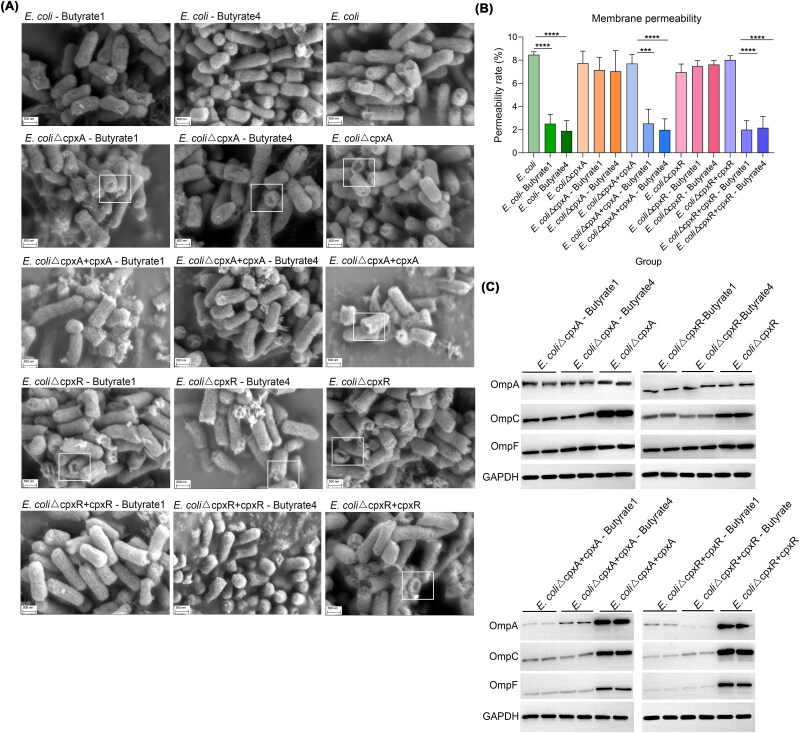
Impact of SCFAs on membrane permeability of knockout and complement strains. SEM images of *cpxA*/*cpxR* knockout and complement strains exposed to butyrate derivatives (A). Quantitative assessment of membrane permeability in genetically modified strains under butyric acid treatment (B). Relative expression profiles of *ompA*/*C*/*F* in knockout and complement strains following butyrate exposure (C).

SEM imaging revealed intact membranes in butyrate-treated wild-type and complemented strains, contrasting with porous membrane structures in knockouts and controls (Fig. 8A). Quantitative PI staining analysis confirmed significantly reduced membrane permeability in butyrate-treated wild-type and complemented strains compared to untreated controls (Fig. 8B). These findings, combined with the observed reduction in bacterial density, demonstrated that butyrate treatment effectively suppresses ARGs dissemination through dual mechanisms of membrane stabilization and growth inhibition.

In addition, this structural preservation correlated with reduced OMP gene expression, suggesting CpxAR activation by butyrate reinforces membrane integrity while occluding conjugation pores. Protein quantification confirmed coordinated CpxAR-dependent suppression of OmpA/C/F in complemented strains (Fig. 8C), linking signal transduction to physical barrier formation against plasmid exchange. The CpxAR system emerges as the central conduit through which aryl-substituted butyrate inhibited ARGs transfer, coupling transcriptional repression of porins with membrane stabilization to block conjugation channels. This dual regulatory-mechanical mechanism highlights the potential of targeting conserved stress response pathways to disrupt resistance gene flux without compromising membrane integrity. Future studies should validate this axis in complex gut communities and explore whether structural analogs of 4-(4-methoxyphenyl)/4-(4-chlorophenyl) butyric acid can be optimized for clinical translation.

## Discussion

The structural stability of gut microbiota constitutes a critical determinant in modulating the dissemination of ARGs. Phylogenetic analysis identifies *Firmicutes* and *Proteobacteria* as predominant contributors to the resistome expansion, particularly through their association with mobile genetic elements [20]. The structural dynamics of gut microbiota significantly modulate intestinal metabolite profiles, which in turn exert bidirectional regulatory effects on the horizontal transfer of ARGs [36]. These microbial-derived metabolites orchestrate ARGs dissemination through dual mechanisms: direct modulation of bacterial physiology and indirect alteration of microenvironmental conditions. Nutritional metabolites may facilitate bacterial proliferation, thereby creating favorable conditions for ARGs transmission through increased microbial density and conjugation opportunities. Conversely, antimicrobial metabolites can suppress bacterial viability, effectively limiting the ecological niches available for resistant strains [37]. Particularly significant are beneficial metabolites that selectively promote commensal flora growth, establishing competitive exclusion against resistant pathogens through ecological niche occupation. Emerging evidence further suggests that metabolites may interfere with bacterial communication systems to regulate conjugation frequency.

These mechanistic insights into microbial ecology raise an important translational question: Can targeted modulation of gut microbiota composition effectively mitigate ARGs dissemination? To address this knowledge gap, we implemented a controlled dietary intervention examining high fiber mediated microbial restructuring. Our findings demonstrate three key effects of sustained high-fiber intake: First, microbial community restructuring manifested as enrichment of beneficial symbionts concurrent with marked depletion of ARG-prone *Enterobacteriaceae*, which may further limit HGT opportunities given the well-documented density-dependence of conjugation efficiency [38]. Second, metabolic reprogramming induced substantial accumulation of SCFAs, particularly butyrate derivatives. Third, these metabolic changes were mechanistically linked to CpxAR-mediated downregulation of conjugative transfer genes (*trfAp* and *trbBp*) and altered membrane permeability. This tripartite mechanism establishes that dietary fiber intervention disrupts ARGs transmission through both ecological competition and molecular regulation.

The temporal dynamics of dietary components critically determine their impact on gut microbial ecosystems. Dietary fiber exerts its influence through two sequential mechanisms: initially as physical substrates shaping microbial habitats, then as metabolic precursors driving functional adaptation. In the colonization phase, fiber provides growth substrates that selectively expand specialized degraders [39], thereby restructuring community composition. During the metabolic phase, colonic anaerobic bacteria (e.g. *Butyrivibrio* spp., *Eubacterium* spp.) activate enzymatic cascades to ferment fiber into SCFAs [40], with butyrate playing dual structural roles: maintaining epithelial [41] and regulating oxygen homeostasis through β-oxidation-driven oxygen [42]. This oxygen-depleted environment creates a self-reinforcing cycle favoring butyrate producers while suppressing oxygen-tolerant *Proteobacteria*. Our experimental data reveal how high-fiber diets amplify this feedforward loop: SCFA-driven niche modification concurrently reduces *Enterobacteriaceae* abundance (ARGs reservoirs [34, 43] and alleviates their pro-inflammatory effects [44]. At cellular level, these ecological shifts decrease bacterial conjugation efficiency by reducing ARGs host populations and altering membrane permeability, ultimately diminishing ARGs transmission risks.

The niche modification mediated by SCFAs not only restructures microbial ecosystems but fundamentally rewires bacterial membrane functionality. Studies have revealed that conjugation mechanisms, although evolutionarily conserved across bacteria [45], are critically dependent on membrane permeability regulated by OMPs. Our integrated transcriptomic, qPCR, and SEM analyses demonstrate that butyric acid derivatives selectively suppress OMP genes in donor *E. coli* (*ompA*, *ompF*) while exerting limited effects on recipient *K. pneumoniae* (partial *ompF*/*fadL* inhibition). This donor-predominant suppression operates through interconnected membrane perturbations: By compromising OmpA-dependent pore formation critical for plasmid channel assembly [46], the derivatives structurally destabilize conjugation infrastructure. Concurrently, co-inhibition of *OmpF* (*r* = 0.82 with conjugation efficiency) [47] and *FadL* disrupts membrane permeability, impeding both small molecule exchange (nutrients/antibiotics) [48] and macromolecular DNA transfer. These molecular alterations manifest phenotypically as enhanced membrane integrity (Fig. 6E), physically restricting the plasticity required for pilus-mediated conjugation [46]. Furthermore, *OmpF* deficiency-induced limitation of SCFAs influx [49] establishes a feedback loop that amplifies membrane homeostasis imbalance, collectively disfavoring conjugation-competent states. These findings collectively position OMP networks as environmental biosensors, prompting a pivotal inquiry: Through what regulatory architectures do extrinsic signals synchronize OMP dynamics to gatekeep ARGs dissemination?

The identified suppression of the CpxAR, a membrane stress sentinel governing OMP homeostasis [50], provides mechanistic clarity to this regulatory puzzle. Through multi-omics integration, we reveal the CpxAR-OMP axis as a signaling circuit through which butyrate derivatives disrupt ARGs propagation via membrane-centric interventions. These compounds (pH 5.6–6.6) subvert conjugation efficiency through CpxAR-mediated membrane remodeling that imposes dual physical and transcriptional barriers to plasmid transfer. Three paradigm-shifting insights emerge from this regulatory architecture. The donor-specific suppression of CpxAR was identified as a previously unrecognized SCFA-responsive conjugation modulator, evidenced by knockout-complementation studies showing complete epistasis: genetic ablation of CpxAR nullified butyrate’s effects on both OMP downregulation and conjugation inhibition, whereas complementation restored wild-type responses. The mechanism further reveals butyrate’s sophisticated membrane sabotage strategy, combining transcriptional suppression of pore-forming OmpF/OmpA proteins with SEM-validated structural reinforcement of membrane integrity that physically obstructs DNA transfer. This multilayered regulatory decoding not only repositions CpxAR as biosensor coordinating microbial stress adaptation and HGT but also illuminates fundamental principles of how gut microbiota leverage nutritional metabolites to enforce population-level control over resistance dissemination.

Collectively, these findings establish the SCFAs-CpxAR-OMP axis as a key biosensory mechanism for containment of resistance gene propagation, providing a biochemical blueprint to design gut microbiome interventions that harness this native signaling pathway to disrupt plasmid transmission while preserving microbial ecological equilibrium. CpxAR acts as the pivotal signaling hub, translating SCFAs-induced stress into transcriptional repression of outer membrane porins (OmpF/C/A) while concurrently stabilizing membrane architecture. This dual functionality creates a biophysical barrier: porin downregulation limits conjugation pore formation, while membrane consolidation reduces permeability to extracellular DNA exchange. These findings highlight the potential for targeted SCFA supplementation as a microbiome-based strategy to curb antimicrobial resistance spread. Our study develops dietary strategies to concurrently mitigate clinical AMR risks and environmental ARGs pollution through competitive exclusion, metabolic interference, membrane engineering, and “gut-environment” cycle break, which bridges gut ecosystem dynamics with implementable One-Health solutions.

## Supplementary Material

SFig1_wraf156

SFig2_wraf156

Supplementary_materials_wraf156

## Data Availability

All data used to support the results of this study, including metagenomic data (BioProject ID: PRJNA879978) and 16S rRNA gene sequencing data (BioProject ID: PRJNA880442), have been uploaded to the NCBI database.
