# Supplementary figures and images for: Decoding the SCFA-CpxAR-OMP axis as a dietary checkpoint against antimicrobial resistance transmission across gut-environment interfaces
Source: ISME J. 2025 Jul 30;19(1):wraf156. doi: 10.1093/ismejo/wraf156 (PMC12416819; doi:10.1093/ismejo/wraf156)

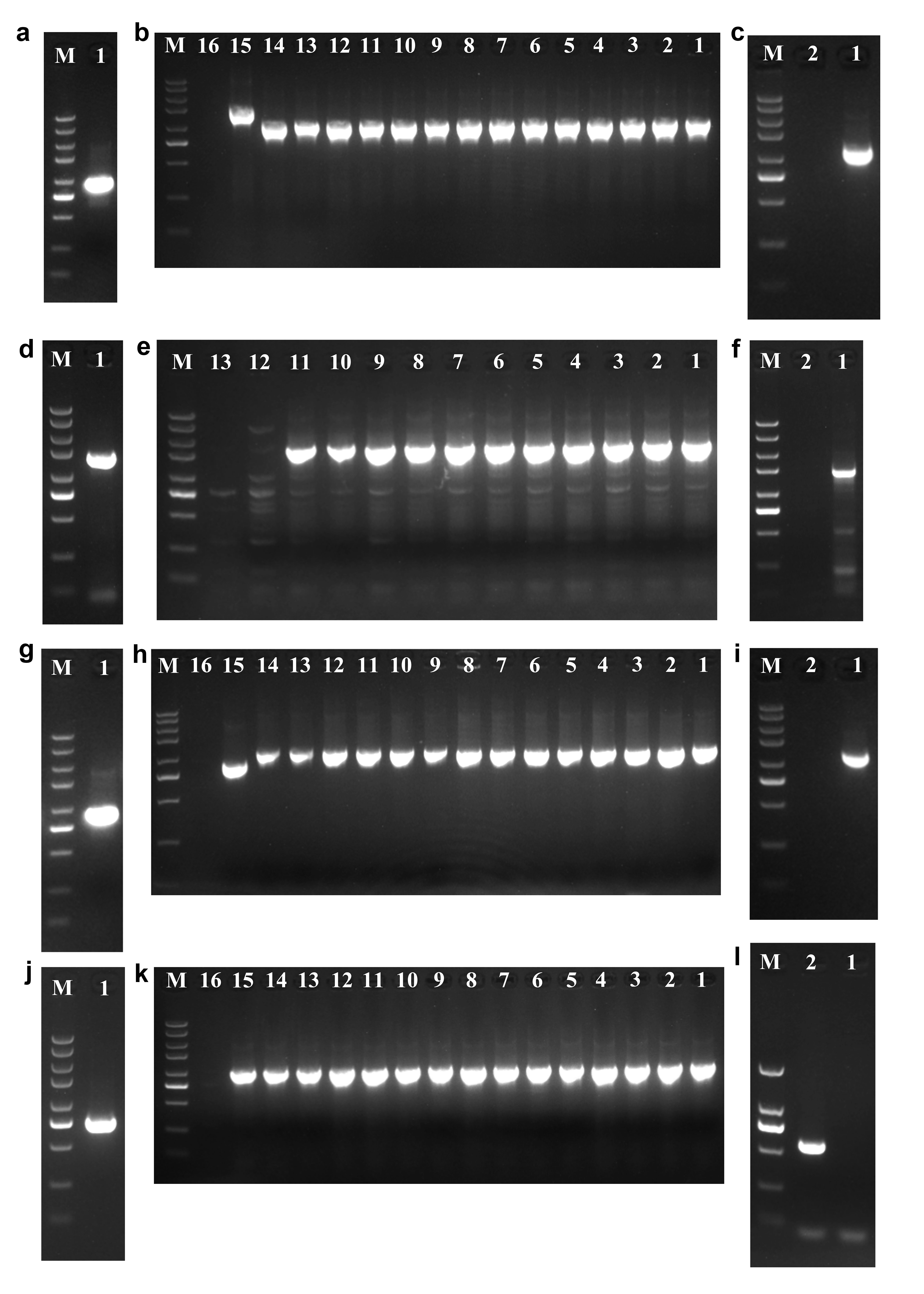

Supplement: SFig1_wraf156 [file sfig1_wraf156.jpeg]

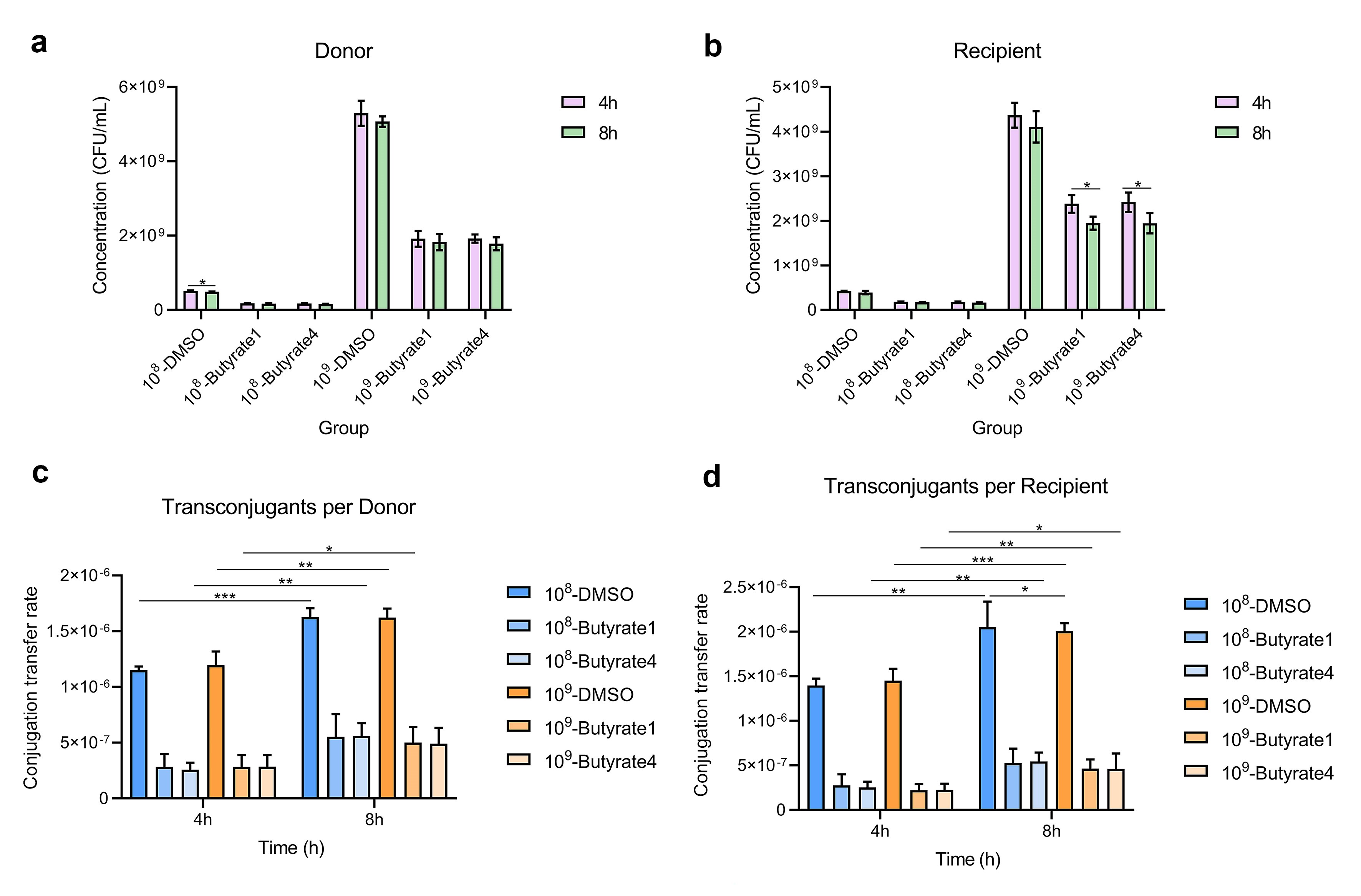

Supplement: SFig2_wraf156 [file sfig2_wraf156.jpeg]
