# Supplementary material for: Decoding the SCFA-CpxAR-OMP axis as a dietary checkpoint against antimicrobial resistance transmission across gut-environment interfaces
Source: ISME J. 2025 Jul 30;19(1):wraf156. doi: 10.1093/ismejo/wraf156 (PMC12416819; doi:10.1093/ismejo/wraf156)
Supplement: Supplementary_materials_wraf156 [file supplementary_materials_wraf156.docx]

**Supplementary Materials and methods**

**M1. Flow cytometric analysis of individual bacterial cells**

Fecal suspensions were passed through 70 μm and 10 μm filters to isolate fecal microbiota samples. The fecal microbiota samples were combined with the donor strain *E. coli*-lacIq-RP4-Tet^R^Km^R^ and incubated at 37°C for 6-8 h. After incubation, the bacterial suspension was diluted with phosphate buffered saline (PBS) to a concentration of 10^7^ cells/ml and analyzed using a flow cytometer (Bio-Rad ZE5, California, USA). An FL1 channel (488 nm filter) The analysis was carried out using a flow cytometer (Bio-Rad ZE5, California, USA) through FL1 channel (equipped with a 488-nm filter) [[1](#_ENREF_1)] which was specifically selected to measure the green fluorescence signal emitted by the receptor bacteria that had received the RP4 plasmid in the intestine. To eliminate the interference of debris and background noise, the analysis was performed using the joint gate setting of forward and lateral scattered light. Finally, the conjugation ratio was calculated based on the number of transconjugants present in the fecal samples.

**M2. Preparation of outer membrane protein antibodies**

First, 20 mg of keyhole limpet hemocyanin (KLH) was dissolved in 2 mL of a 5 mM EDTA aqueous solution. Separately, 8 mg of sulfo-SMCC was fully dissolved in 50 μL of dimethyl sulfoxide (DMSO), followed by the addition of 150 μL of PBS and thorough mixing. The sulfo-SMCC solution was then added dropwise to the KLH solution and gently mixed. The activated KLH solution was dialyzed with magnetic stirring at 4°C for 1 h, followed by 2h dialysis sessions in fresh PBS, and the process was repeated. The activated and dialyzed KLH was stored at 4°C. Next, 4 mg of the peptide was dissolved in 50 μL of DMSO, and 200 μL of PBS was added and mixed quickly. KLH was added at a peptide-to-KLH ratio of 1 mg:680 μg. The mixture was allowed to react at room temperature for 2 h, then dialyzed with magnetic stirring at 4°C overnight, and finally stored at -20°C. New Zealand rabbits were immunized via multiple subcutaneous injections, with 0.2 mL administered at each injection point. The initial immunization dose was 0.1-1.0 mg, and the antigen dose for the second and fourth immunizations was reduced by half. Seven days after immunization, a small serum sample was collected from the middle ear artery of the animals for testing. After the test, whole blood was collected. Once the blood had settled into layers, the supernatant was centrifuged at 12,000 rpm for 2 min. Then, 50 mL of the supernatant was mixed with 100 μL of a 10% sodium thiomersal solution to achieve a final concentration of 0.02% and stored at -20°C. The serum was subjected to ELISA testing. Qualified serum was filtered through a 0.45 μm microporous membrane and then processed through multiple chromatography steps. The collected antibodies were concentrated and dialyzed multiple times at room temperature. The concentration of the antibodies was measured using a DeNovix DS-11 ultra-micro spectrophotometer (Thermo Fisher Scientific, USA) to obtain purified antibodies [[2](#_ENREF_2)].

**M3. Western Blot**

The donor strain *E. coli* was incubated with butyric acid at 37°C for 8 hr. Then, 10 mL of the bacterial sample was collected by centrifugation at 6000 rpm. The collected bacteria were resuspended in 1 mL of RIPA lysis solution and lysed at 4°C for 30 minutes. After lysis, the sample was centrifuged at 12000 rpm and 4°C for 10 min. The protein concentration in the extracted supernatant was quantified using the BCA protein assay. An 80 μg aliquot of the total protein was mixed with 5x protein loading buffer, thoroughly vortexed, and then denatured in boiling water for 10 minutes. The denatured protein sample was subjected to electrophoresis at 140 V until the loading dye had completely migrated through the separation gel, at which point the electrophoresis was stopped. The gel was carefully removed and equilibrated in the prepared 1x membrane transfer solution for 20 min. A PVDF membrane that was cut to an appropriate size was first activated with methanol and then equilibrated with thin filter paper in the transfer solution for 20 min. The transfer sandwich was assembled in the following order: negative electrode, thin filter paper, PVDF membrane, thin filter paper, and positive electrode. A constant-current transfer of 200 mA was set and maintained for 1 h. A 5% skim milk powder solution in 1x TBST was prepared for blocking the membrane. The primary antibody was added at a dilution of 1:1000, and the membrane was incubated overnight on a shaking bed at 4°C. After incubation, the membrane was washed three times with 1x TBST. The washed membrane was then placed in a diluted secondary antibody solution (1:3000) and incubated at room temperature on a shaking table for 1 h. Subsequently, the membrane was rinsed thrice with 1x TBST. The liquid on the PVDF membrane was carefully drained, and the ECL working solution was evenly dropped onto the membrane. The membrane was incubated at room temperature for 2 min. Excess ECL solution was removed, and the membrane was placed in a fully automatic chemiluminescence image analysis system for imaging and photography. The grayscale values of the bands were analyzed using the Gelpro32 grayscale analysis software [[3](#_ENREF_3)].

**M4. Scanning Electron Microscope (SEM)**

The donor bacterium *E. coli* was incubated with butyric acid at 37°C for 8 hours. Subsequently, the bacterial cells were harvested by centrifugation at 6000 rpm. The collected cells were then fixed by adding 2.5% glutaraldehyde and incubating at 4°C for 24 h. After the fixation step, the cells were centrifuged again at 6000 rpm. The supernatant was discarded, and the cells were washed thrice with PBS. Next, a gradient dehydration process was carried out. The cells were sequentially treated with 30%, 50%, 70%, 80%, 90%, 95%, and 100% anhydrous ethanol. They were dehydrated twice with 100% anhydrous ethanol, with each dehydration step lasting for 10 min. Afterward, the cells were centrifuged at 6000 rpm for 3 min. Finally, the cells were freeze-dried using a freeze dryer. The status of the bacterial cell membrane was observed using a Zeiss Supra Gemini series 35V scanning electron microscope (Zeiss, Germany) [[4](#_ENREF_4)].

‌**M5. Bacterial Membrane Permeability Assay**‌

To evaluate membrane integrity, bacterial suspensions treated with butyrate derivatives were incubated with propidium iodide (PI, 1 μg/mL) for 15 min at 37°C in the dark. PI, a membrane-impermeant fluorescent dye, selectively enters cells with compromised membranes and intercalates into DNA. After washing twice with PBS to remove unbound dye, fluorescence intensity (excitation: 530 nm; emission: 610 nm) was quantified using a microplate reader (SpectraMax M5). Untreated bacteria and PBS-only wells served as negative and blank controls, respectively. Membrane permeability was expressed as the relative fluorescence units (RFU) normalized to the negative control and a positive control.

**Supplementary Table1. Concentrations of antibiotics and metabolites used in the experiments**

| Name | Concentration |
| --- | --- |
| Tetracycline | 50mg/L |
| Kanamycin | 60mg/L |
| Chloramphenicol | 40mg/L |
| Meropenem | 2.25mg/L |
| Cellulose | 100mg/mL |
| 4-hydroxybutyric acid | 23mM |
| 3-hydroxy-3-methylbutyric acid | 23mM |
| 4-(4-chlorophenyl) butyric acid | 23mM |
| 4-(4-methoxyphenyl) butyric acid | 23mM |

**Supplementary File1. CpxA/CpxR sequence information of Escherichia coli two-component system**

**CpxA:**

ATGATAGGCAGCTTAACCGCGCGCATCTTCGCCATCTTCTGGCTGACGCTGGCGCTGGTGTTGATGTTGGTTTTGATGTTACCCAAGCTCGATTCACGCCAGATGACCGAGCTTCTGGATAGCGAACAGCGTCAGGGGCTGATGATTGAGCAGCATGTCGAAGCGGAGCTGGCGAACGATCCGCCCAACGATTTAATGTGGTGGCGGCGTCTGTTCCGGGCGATTGATAAGTGGGCACCGCCAGGACAGCGTTTGTTATTGGTGACCACCGAAGGCCGCGTGATCGGCGCTGAACGCAGCGAAATGCAGATCATTCGTAACTTTATTGGTCAGGCCGATAACGCCGATCATCCGCAGAAGAAAAAGTATGGCCGCGTGGAACTGGTCGGTCCGTTCTCCGTGCGTGATGGCGAAGATAATTACCAACTTTATCTGATTCGTCCGGCCAGCAGTTCTCAATCCGATTTCATTAACTTACTGTTTGACCGCCCGCTATTACTGCTGATTGTCACCATGTTGGTCAGTACGCCGCTGCTGTTGTGGTTGGCCTGGAGTCTGGCAAAACCGGCGCGTAAGCTGAAAAACGCTGCCGATGAAGTTGCCCAGGGAAACTTACGCCAGCACCCGGAACTGGAAGCGGGGCCACAGGAATTCCTTGCCGCAGGTGCCAGTTTTAACCAGATGGTCACCGCGCTGGAGCGCATGATGACCTCTCAGCAGCGTCTGCTTTCTGATATCTCTCACGAGCTGCGCACCCCGCTGACGCGTCTGCAACTGGGTACGGCGTTACTGCGCCGTCGTAGCGGTGAAAGCAAGGAACTGGAGCGTATTGAAACCGAAGCGCAACGTCTGGACAGCATGATCAACGATCTGTTGGTGATGTCACGTAATCAGCAAAAAAACGCGCTGGTTAGCGAAACCATCAAAGCCAACCAGTTGTGGAGTGAAGTGCTGGATAACGCGGCGTTCGAAGCCGAGCAAATGGGCAAGTCGTTGACAGTTAACTTCCCGCCTGGGCCGTGGCCGCTGTACGGCAATCCGAACGCCCTGGAAAGTGCGCTGGAAAACATTGTTCGTAATGCTCTGCGTTATTCCCATACGAAGATTGAAGTGGGCTTTGCGGTAGATAAAGACGGTATCACCATTACGGTGGACGACGATGGTCCTGGCGTTAGCCCGGAAGATCGCGAACAGATTTTCCGTCCGTTCTATCGTACCGATGAAGCACGCGATCGTGAATCTGGCGGTACAGGTTTGGGGCTGGCGATTGTTGAAACCGCCATTCAGCAGCATCGTGGCTGGGTGAAGGCAGAAGACAGCCCGCTGGGCGGTTTACGGCTGGTGATTTGGTTGCCGCTGTATAAGCGGAGTTAA

**CpxR:**

ATGAATAAAATCCTGTTAGTTGATGATGACCGAGAGCTGACTTCCCTATTAAAGGAGCTGCTCGAGATGGAAGGCTTCAACGTGATTGTTGCCCACGATGGGGAACAGGCGCTTGATCTTCTGGACGACAGCATTGATTTACTTTTGCTTGACGTAATGATGCCGAAGAAAAATGGTATCGACACATTAAAAGCACTTCGCCAGACACACCAGACGCCTGTCATTATGTTGACGGCGCGCGGCAGTGAACTTGATCGCGTTCTCGGCCTTGAGCTGGGCGCAGATGACTATCTCCCGAAACCGTTTAATGATCGTGAGCTGGTGGCACGTATTCGCGCGATCCTGCGCCGTTCGCACTGGAGCGAGCAACAGCAAAACAACGACAACGGTTCACCGACACTGGAAGTTGATGCCTTAGTGCTGAATCCAGGCCGTCAGGAAGCCAGCTTCGACGGGCAAACGCTGGAGTTAACCGGTACTGAGTTTACCCTGCTCTATTTGCTGGCACAGCATCTGGGTCAGGTGGTTTCCCGTGAACATTTAAGCCAGGAAGTGTTGGGCAAACGCCTGACGCCTTTCGACCGCGCTATTGATATGCACATTTCCAACCTGCGTCGTAAACTGCCGGATCGTAAAGATGGTCACCCGTGGTTTAAAACCTTGCGTGGTCGCGGCTATCTGATGGTTTCTGCTTCATGA

**Supplementary File2. E. coli outer membrane protein sequence information**

**OmpF:**

TGGGCCGCAGGGTGCGGCGCGCGTCTGCACCGCTGCAGAATCTATAACAAAGATGGCAACAAAGTAGATCTGTACGGTAAAGCTGTTGGTCTGCATTATTTTTCCAAGGGTAACGGTGAAAACAGTTACGGTGGCAATGGCGACATGACCTATGCCCGTCTTGGTTTTAAAGGGGAAACTCAAATCAATTCCGATCTGACCGGTTATGGTCAGTGGGAATATAACTTCCAGGGTAACAACTCTGAAGGCGCTGACGCTCAAACTGGTAACAAAACGCGTCTGGCATTCGCGGGTCTTAAATACGCTGACGTTGGTTCTTTCGATTACGGCCGTAACTACGGTGTGGTTTATGATGCACTGGGTTACACCGATATGCTGCCAGAATTTGGTGGTGATACTGCATACAGCGATGACTTCTTCGTTGGTCGTGTTGGCGGCGTTGCTACCTATCGTAACTCCAACTTCTTTGGTCTGGTTGATGGCCTGAACTTCGCTGTTCAGTACCTGGGTAAAAACGAGCGTGACACTGCACGCCGTTCTAACGGCGACGGTGTTGGCGGTTCTATCAGCTACGAATACGAAGGCTTTGGTATCGTTGGTGCTTATGGTGCAGCTGACCGTACCAACCTGCAAGAAGCTCAACCTCTTGGCAACGGTAAAAAAGCTGAACAGTGGGCTACTGGTCTGAAGTACGACGCGAACAACATCTACCTGGCAGCGAACTACGGTGAAACCCGTAACGCTACGCCGATCACTAATAAATTTACAAACACCAGCGGCTTCGCCAACAAAACGCAAGACGTTCTGTTAGTTGCGCAATACCAGTTCGATTTCGGTCTGCGTCCGTCCATCGCTTACACCAAATCTAAAGCGAAAGACGTAGAAGGTATCGGTGATGTTGATCTGGTGAACTACTTTGAAGTGGGCGCAACCTACTACTTCAACAAAAACATGTCCACCTATGTTGACTACATCATCAACCAGATCGATTCTGACAACAAACTGGGCGTAGGTCAGACGACACCCGTCAATC

WAAGCGARLHRCRIYNKDGNKVDLYGKAVGLHYFSKGNGENSYGGNGDMTYARLGFKGETQINSDLTGYGQWEYNFQGNNSEGADAQTGNKTRLAFAGLKYADVGSFDYGRNYGVVYDALGYTDMLPEFGGDTAYSDDFFVGRVGGVATYRNSNFFGLVDGLNFAVQYLGKNERDTARRSNGDGVGGSISYEYEGFGIVGAYGAADRTNLQEAQPLGNGKKAEQWATGLKYDANNIYLAANYGETRNATPITNKFTNTSGFANKTQDVLLVAQYQFDFGLRPSIAYTKSKAKDVEGIGDVDLVNYFEVGATYYFNKNMSTYVDYIINQIDSDNKLGVGQTTPVN

**OmpA:**

CTTCAGCCGATGCCTGGATCGCTACCGTAGCGCAGCCGCTCCGAAAGATAACACCTGGTACACTGGTGCTAAACTGGGCTGGTCCCAGTACCATGACACTGGTTTCATCAACAACAATGGCCCGACCCATGAAAACCAACTGGGCGCTGGTGCTTTTGGTGGTTACCAGGTTAACCCGTATGTTGGCTTTGAAATGGGTTACGACTGGTTAGGTCGTATGCCGTACAAAGGCAGCGTTGAAAACGGTGCATACAAAGCTCAGGGCGTTCAACTGACCGCTAAACTGGGTTACCCAATCACTGACGACCTGGACATCTACACTCGTCTGGGTGGCATGGTATGGCGTGCAGACACTAAATCCAACGTTTATGGTAAAAACCACGACACCGGCGTTTCTCCGGTCTTCGCTGGCGGTGTTGAGTACGCGATCACTCCTGAAATCGCTACCCGTCTGGAATACCAGTGGACCAACAACATCGGTGACGCACACACCATCGGCACTCGTCCGGACAACGGCATGCTGAGCCTGGGTGTTTCCTACCGTTTCGGTCAGGGCGAAGCAGCTCCAGTAGTTGCTCCGGCTCCAGCTCCGGCACCGGAAGTACAGACCAAGCACTTCACTCTGAAGTCTGACGTTCTGTTCAACTTCAACAAAGCAACCCTGAAACCGGAAGGTCAGGCTGCTCTGGATCAGCTGTACAGCCAGCTGAGCAACCTGGATCCGAAAGACGGTTCCGTAGTTGTTCTGGGTTACACCGACCGCATCGGTTCTGACGCTTACAACCAGGGTCTGTCCGAGCGCCGTGCTCAGTCTGTTGTTGATTACCTGATCTCCAAAGGTATCCCGGCAGACAAGATCTCCGCACGTGGTATGGGCGAATCCAACCCGGTTACTGGCAACACCTGTGACAACGTGAAACAGCGTGCTGCACTGATCGACTGCCTGGCTCCGGATCGTCGCGTAGAGATCGAAGGTAGAAGGAACCCAGGTTCGCC

FSRCLDRYRSAAAPKDNTWYTGAKLGWSQYHDTGFINNNGPTHENQLGAGAFGGYQVNPYVGFEMGYDWLGRMPYKGSVENGAYKAQGVQLTAKLGYPITDDLDIYTRLGGMVWRADTKSNVYGKNHDTGVSPVFAGGVEYAITPEIATRLEYQWTNNIGDAHTIGTRPDNGMLSLGVSYRFGQGEAAPVVAPAPAPAPEVQTKHFTLKSDVLFNFNKATLKPEGQAALDQLYSQLSNLDPKDGSVVVLGYTDRIGSDAYNQGLSERRAQSVVDYLISKGIPADKISARGMGESNPVTGNTCDNVKQRAALIDCLAPDRRVEIEGRRNPGS

**OmpC:**

GGGACAGCTCTGCTGGTAGCAGGCGCAGCAAACGCTGCTGAAGTTTACAACAAAGACGGCAACAAATTAGATCTGTACGGTAAAGTAGACGGCCTGCACTATTTCTCTGACAACAAAGATGTAGATGGCGACCAGACCTACATGCGTCTTGGCTTCAAAGGTGAAACTCAGGTTACTGACCAGCTGACCGGTTACGGCCAGTGGGAATATCAGATCCAGGGCAACAGCGCTGAAAACGAAAACAACTCCTGGACCCGTGTGGCATTCGCAGGTCTGAAATTCCAGGATGTGGGTTCTTTCGACTACGGTCGTAACTACGGCGTTGTTTATGACGTAACTTCCTGGACCGACGTACTGCCAGAATTCGGTGGTGACACCTACGGTTCTGACAACTTCATGCAGCAGCGTGGTAACGGCTTCGCGACCTACCGTAACACTGACTTCTTCGGTCTGGTTGACGGCCTGAACTTTGCTGTTCAGTACCAGGGTAAAAACGGCAACCCATCTGGTGAAGGCTTTACTAGTGGCGTAACTAACAACGGTCGTGACGCACTGCGTCAAAACGGCGACGGCGTCGGCGGTTCTATCACTTATGATTACGAAGGTTTCGGTATCGGTGGTGCGATCTCCAGCTCCAAACGTACTGATGCTCAGAACACCGCTGCTTACATCGGTAACGGCGACCGTGCTGAAACCTACACTGGTGGTCTGAAATACGACGCTAACAACATCTACCTGGCTGCTCAGTACACCCAGACCTACAACGCAACTCGCGTAGGTTCCCTGGGTTGGGCGAACAAAGCACAGAACTTCGAAGCTGTTGCTCAGTACCAGTTCGACTTCGGTCTGCGTCCGTCCCTGGCTTACCTGCAGTCTAAAGGTAAAAACCTGGGTCGTGGCTACGACGACGAAGATATCCTGAAATATGTTGATGTTGGTGCTACCTACTACTTCAACAAAAACATGTCCACCTACGTTGACTACAAAATCAACCTGCTGGACGACAACCAGTTCACTCGTGACGCTGGCATCAACACTGATAACATCCGTCCCAC

GTALLVAGAANAAEVYNKDGNKLDLYGKVDGLHYFSDNKDVDGDQTYMRLGFKGETQVTDQLTGYGQWEYQIQGNSAENENNSWTRVAFAGLKFQDVGSFDYGRNYGVVYDVTSWTDVLPEFGGDTYGSDNFMQQRGNGFATYRNTDFFGLVDGLNFAVQYQGKNGNPSGEGFTSGVTNNGRDALRQNGDGVGGSITYDYEGFGIGGAISSSKRTDAQNTAAYIGNGDRAETYTGGLKYDANNIYLAAQYTQTYNATRVGSLGWANKAQNFEAVAQYQFDFGLRPSLAYLQSKGKNLGRGYDDEDILKYVDVGATYYFNKNMSTYVDYKINLLDDNQFTRDAGINTDNIRP

The part marked in blue is consistent with the data base sequence, and an antigen is designed for that area

**Figure legends**

**Supplementary Figure 1. Electrophoretic maps of *cpxA* and *cpxR* gene knockout and replenishment processes in *Escherichia coli*.**

Target fragment of *cpxA*, 1: *cpxA* gene target fragment, 960bp (**a**). Knockout gene positive clone screening, 1-14: amplification results of clones 1-14; 15: amplification results of the original strain; 16: no template negative control amplification result (**b**). Knockout strain clone (*cpxA)* validation, 1: amplification result of the first clone, 2: amplification result without template negative control (**c**). Recombinant plasmid construction, 1: amplification result of *cpxA* (**d**). Positive clones of *cpxA* recombinant plasmid, 1-12: amplification results of clones 1-12; 13: no template negative control (**e**). Amplification results of *cpxA* complement strain, 1: amplification results of monoclonal culture medium; 2: The amplification result of *cpxA* complement strain (**f**). Target fragment of *cpxR*, 1: *cpxR* gene target fragment, 960bp (**g**). Knockout gene positive clone screening, 1-14: amplification results of clones 1-14; 15: amplification results of the original strain; 16: no template negative control amplification result (**h**). Knockout strain clone (*cpxR*), 1: amplification result of the first clone, 2: amplification result without template negative control (**i**). Recombinant plasmid construction, 1: amplification result of *cpxR* (**j**). Positive clones of *cpxR* recombinant plasmid, 1-12: amplification results of clones 1-12; 13: no template negative control (**k**). Amplification results of *cpxR* complement strain, 1: amplification results of monoclonal culture medium; 2: The amplification result of *cpxR* complement strain (**l**). All M are DNA molecular weight standards, with molecular weights from top to bottom being 5000, 3000, 2000, 1500, 1000, 750, 500, 250, and 100bp, with 750bp highlighted.

**Supplementary Figure 2.‌ Time-dependent changes in donor and recipient bacterial densities during conjugation transfer.** Donor bacterial densities (10⁸ and 10⁹ CFU/mL) under different butyric acid conditions at 4 h and 8 h (**a**);‌ Recipient bacterial densities (10⁸ and 10⁹ CFU/mL) under different butyric acid conditions at 4 h and 8 h (**b**); ‌ Conjugation transfer rate (transconjugants per donor) across time points and bacterial densities (**c**); ‌ Conjugation transfer rate (transconjugants per recipient) across time points and bacterial densities (**d**).

**Reference**

1. Xue Z, Sendamangalam VR, L. GC *et al.* Multiple roles of extracellular polymeric substances on resistance of biofilm and detached clusters. *Environ Sci Technol* 2012;**46**:13212-9. https://doi.org/10.1021/es3031165

2. Yang L, Niu K, Wang J *et al.* Nucleolin lactylation contributes to intrahepatic cholangiocarcinoma pathogenesis via RNA splicing regulation of MADD. *J Hepatol* 2024;**81**:651-66. https://doi.org/10.1016/j.jhep.2024.04.010

3. Wang E, Qin Z, Yu Z *et al.* Molecular characterization, phylogenetic, expression, and protective immunity analysis of OmpF, a promising candidate immunogen against *Yersinia ruckeri* infection in channel catfish. *Front Immunol* 2018;**9**:2003. https://doi.org/10.3389/fimmu.2018.02003

4. Shahryari S, Zahiri HS, Haghbeen K *et al.* High phenol degradation capacity of a newly characterized *Acinetobacter* sp. Sa01: Bacterial cell viability and membrane impairment in respect to the phenol toxicity. *Ecotoxicol Environ Saf* 2018;**164**:455-66. https://doi.org/10.1016/j.ecoenv.2018.08.051
